# VEGF mitigates histone‐induced pyroptosis in the remote liver injury associated with renal allograft ischemia–reperfusion injury in rats

**DOI:** 10.1111/ajt.14699

**Published:** 2018-03-23

**Authors:** Hailin Zhao, Han Huang, Azeem Alam, Qian Chen, Ka Chuen Suen, Jiang Cui, Qizhe Sun, Rele Ologunde, Wenwen Zhang, Qingquan Lian, Daqing Ma

**Affiliations:** ^1^ Anaesthetics, Pain Medicine and Intensive Care Department of Surgery and Cancer Faculty of Medicine Imperial College London Chelsea & Westminster Hospital London UK; ^2^ Department of Anaesthesiology Southwest Hospital Third Military Medical University Chongqing China; ^3^ Department of Anaesthesiology West China Second University Hospital Sichuan University Chengdu China; ^4^ The Second Affiliated Hospital Wenzhou Medical University Wenzhou China

**Keywords:** basic (laboratory) research/science, cell death, ischemia–reperfusion injury (IRI), kidney transplantation/nephrology

## Abstract

Clinical evidence has indicated a possible link between renal injury and remote liver injury. We investigated whether extracellular histone mediates remote hepatic damage after renal graft ischemia–reperfusion injury, while vascular endothelial growth factor (VEGF) is protective against remote hepatic injury. In vitro, hepatocyte HepG2 cultures were treated with histone. In vivo, the Brown‐Norway renal graft was stored in 4°C preservation solution for 24 hours and then transplanted into a Lewis rat recipient; blood samples and livers from recipients were harvested 24 hours after surgery. Prolonged cold ischemia in renal grafts enhanced liver injury 24 hours after engraftment. *Caspase‐1, ASC, NLRP3*, and *AIM2* expressions in hepatocyte, CD68^+^‐infiltrating macrophages, tissue, and serum interleukin‐1β and ‐18 were greatly elevated, indicating that pyroptosis occurred in the liver and resulted in acute liver functional impairment. Blocking the caspase‐1 pathway decreased the number of necrotic hepatocytes. VEGF treatment suppressed the hepatocyte pyroptosis and liver function was partially restored. Our data suggested that renal allograft ischemia–reperfusion injury is likely associated with acute liver damage due to hepatocyte pyroptosis induced by histone and such injury may be protected by VEGF administration. VEGF, therefore, may serve as a new strategy against other remote organ injuries related to renal transplantation.

## Abstract 2

Vascular endothelial growth factor mitigates pyroptosis in the liver following renal allograft engraftment.

**Abbreviations**
0.1% PBS‐T: 0.1% Triton in PBS
AIM2: absent in melanoma 2
AKI: acute kidney injury
ALT: alanine aminotransferase
ASC: Apoptosis‐associated speck‐like protein containing a CARD
AST: asparate aminotransferase
DAMP: damage‐associated molecular pattern
FACS: fluorescence‐activated cell sorting
FI: fluorescence intensity
HIF: hypoxia inducible factor
IFN: interferon
IL: interleukin
IRI: ischemia–reperfusion injury
NF‐κB: nuclear factor‐κB
NLRP3: nucleotide‐binding domain, leucine‐rich repeat containing protein 3
TLR: Toll‐like receptor
TUNEL: terminal deoxynucleotidyl transferase dUTP nick end labeling
VEGF: vascular endothelial growth factor

## 1 INTRODUCTION

Ischemia–reperfusion injury (IRI) is an inevitable consequence of renal transplantation and a major determinant of graft survival. Renal IRI is associated with deleterious consequences for several organs.^1^, ^2^ Accumulating clinical evidence has identified a close relationship between renal injury and injuries to other organ systems, including heart, lung, and liver.^3^ Indeed, impaired liver function is often seen in patients with acute kidney injury (AKI).^4^, ^5^ In addition, in‐hospital death is more likely in patients with AKI and liver failure than in those with AKI alone.^6^ The pathophysiology of remote liver injury after AKI is complex and incompletely understood, although both preclinical and clinical studies have shown that inflammation is an important mediator.^2^ The exact functions of the various cytokines involved in regulating the complex inflammatory events after IRI are not yet fully unraveled.^7^

The liver is particularly vulnerable to inflammatory challenge from the distant renal graft because it is abundantly perfused with systematic circulation. Liver injury could develop secondary to delayed graft function in a renal graft recipient. Remote hepatic injury is believed to be initiated and sustained by proinflammatory cytokines that are released or activated during IRI after renal damage.^8^, ^9^ If liver repair and regenerative mechanism are not activated promptly, acute liver functional impairment could occur. Clinical management of such patients is difficult, and effective organ protective strategy is therefore required.

Recently, extracellular histone has been identified as a key inflammatory mediator in renal injury.^10^ Histone is a highly conserved eukaryotic chromosomal protein. Under oxidative or inflammatory stress, histone undergoes translocation from the nucleus to the cytoplasm, and then it is secreted from the necrotic cells.^11^ In the liver, histone has been shown to mediate hepatocyte cell death and inflammation.^12^, ^13^ It is suggested that histone mediates cell death and inflammation by binding to Toll‐like receptors (eg, TLR‐4 and TLR‐9).^14^ Activation of TLRs may trigger the downward cascade including the inflammasome to activate pyroptosis and produce proinflammatory cytokines, exacerbating the injury and causing a systemic response.^15^, ^16^

Pyroptosis is a lytic type of cell death and a form of regulated necrosis that is inherently associated with inflammation and distinguished from other forms of cell death by the associated secretion of interleukin (IL)‐1β after caspase‐1 activation. The cellular processes that occur during pyroptosis include nuclear condensation, DNA damage, cell swelling, and, finally, cell lysis, with the subsequent release of IL‐1β and are reliant on intracellular sensors of bacterial products and formation of the inflammasome. Pyroptosis is a proinflammatory response that is triggered by a variety of pathologic stimuli, including myocardial infarction, stroke, and malignancy. Various studies have indicated that pyroptosis is intrinsically involved in the development of infectious diseases, nervous system disorders, and atherosclerotic processes.^17^, ^18^, ^19^ Pyroptosis is thought to be a key modulator of the immune response to microbial infection, while pathogens that have evolved and developed mechanisms to bypass pyroptosis demonstrate enhanced disease‐causing and septic potential.^20^

To date, there is a lack of studies about the effects of acute renal allograft injury on the liver. In the present study, we tested the hypothesis that IRI in renal allografts would initiate the distant hepatic injury. The underlying molecular mechanism, which is centered on pyroptosis, was also explored in this study.

## 2 MATERIALS AND METHODS

### 2.1 In vitro cell culture

Human hepatocyte HepG2 cells were cultured in EMEM, and human monocyte/macrophage U937 cells (European Cell Culture Collection, Porton Down, UK) were cultured in RPMI 1640 medium. The culture medium was supplemented with 10% FBS (Invitrogen, Carlsbad, CA), 2 mmol/L l‐glutamine (Invitrogen), and 100 U/mL penicillin–streptomycin (Invitrogen).

### 2.2 Cell treatments in vitro

Some cohort cultures of HepG2 cultures were treated with histone H3 recombinant protein (20 μg/mL; Sigma‐Aldrich, Poole, UK), some cohort cultures were treated with VEGF recombinant protein (5 ng⁄mL; Thermo Fisher Scientific, Paisley, UK) or PBS vehicle for 24 hours, and some cohort cultures were treated with human nucleotide‐binding domain, leucine‐rich repeat containing protein 3 (*NLRP3*) siRNA (SI03060323; Qiagen, Crawley, West Sussex, UK), *AIM2* siRNA (SI04261432; Qiagen), *VEGF* siRNA (sc‐29520; Santa Cruz Biotechnology, Dallas, TX), *VEGFR1* siRNA (sc‐29319; Santa Cruz), *VEGFR2* siRNA (sc‐29318; Santa Cruz), or scrambled siRNA (Qiagen) 6 hours before experiments. siRNA was dissolved in siRNA suspension buffer and administered to HepG2 cells at a dose of 20 nmol/L; scrambled siRNA served as a negative control. The transfection was carried out through highly efficient Lipofectamine Transfection Reagent (Thermo Fisher Scientific UK).^21^ Cells were incubated with siRNA for 6 hours before histone treatment. Some cell cohort received caspase‐1 inhibitor Ac‐YVAD‐CHO (20 μmol/L; Santa Cruz).

### 2.3 Flow cytometry

Reactive oxygen species production was monitored by the measurement of superoxide (O^2−^) generation by using the fluorescent dyes dihydroethidium (DHE).^22^ Cells were incubated in DHE (2 μmol/L) for 30 minutes at 37°C in the dark. The cells were washed with PBS. Fluorescence intensity was acquired and analyzed by using flow cytometry (FACSCalibur; Becton Dickinson, Sunnyvale, CA). Each assay included at least 10 000 gated events. Propidium iodide (PI; Sigma Aldrich, St. Louis, MO) staining was used to examine cell death as described. Cells were harvested in a fluorescence‐activated cell sorting (FACS) tube and washed twice before resuspension in FACS buffer. PI was added to make the final concentration to 1 μg/mL and incubated in dark for 5 minutes. PI fluorescence was detected by using flow cytometry.

### 2.4 Renal transplantation

Inbred adult male Brown–Norway rats BN, RT^1n^) and Lewis (LEW, RT1^1^) rats weighing 225 to 250 g were purchased from Harlan, Bicester, UK and bred in temperature‐ and humidity‐controlled cages in a specific pathogen‐free facility at Chelsea‐Westminster Campus, Imperial College London. This study was approved by the Home Office, United Kingdom, and all animal procedures were carried out in accordance with the United Kingdom Animals (Scientific Procedures) Act of 1986. BN‐to‐LEW rat renal transplantation was used. Rat donor kidneys were transplanted orthotopically into recipients by using standard microvascular techniques.^21^ Briefly, the donor's left kidney, aorta, and inferior vena cava were carefully exposed. The aorta was clamped below the renal vessel. An elliptical aortic patch was created. The kidney graft was then extracted, flushed, and stored in 4°C heparinized Soltran Preserving Solution (Baxter Healthcare, Newbury, UK). After the cold ischemia, the recipient's renal artery and vein were carefully isolated and cross‐clamped, the left kidney was extracted, and the donor renal vein was connected to the recipient renal vein through end‐to‐end anastomosis with continuous 8‐0 sutures. The arterial anastomosis between the donor aortic patch was connected to recipient aorta in an end‐to‐side manner. The successful perfusion of renal graft was confirmed by an instant color change of the kidney and rapid expansion of renal arteries and vein. Urinary reconstruction was performed by ureter‐to‐bladder anastomosis. The total surgical ischemia time was restricted to less than 45 minutes. The contralateral native kidney was excised immediately after surgery.

### 2.5 Animal treatment

All recipient animals received Cyclosporine A (5 mg/kg per day through intramuscular injection; Tocris, UK). The animals were administered histone H3 (calf thymus histone H3; Sigma‐Aldrich) via intravenous injection (low dose 10 mg/kg, high dose 50 mg/kg).^23^ VEGF recombinant protein (10 mg/kg intravenous injection; LSBio, Seattle, WA), caspase‐1 inhibitor (ac‐YVAD‐cmk, 1.25 μmol/kg via intravenous injection; Sigma Aldrich), and TLR‐4 inhibitor (TAK‐242, 10 mg/kg via intravenous injection; EMD Millipore UK Ltd., Livingston, UK) were administered to the rat recipients.

### 2.6 Hydrodynamic tail vein injection

Rat VEGF siRNA (Qiagen, SI01994454), rat VEGF R2 (Qiagen, SI01528415), or scrambled siRNAs (negative control) (Qiagen) were dissolved in siRNA suspension buffer and further diluted in RNase‐free PBS before use. siRNA targeting rat VEGF was administered via hydrodynamic tail vein injection according to our established protocol^24^ after transplantation. VEGF or scrambled siRNA (200 μg in 10 mL of PBS) was rapidly injected (within 30 seconds) via a tail vein with the rats under anesthesia.

### 2.7 Hematoxylin–eosin staining

Liver samples obtained at various determination points were fixed in 4% buffered formalin and then embedded in paraffin, in accordance with standard procedures. Sections (5 μm) were stained with hematoxylin–eosin and examined microscopically. All samples were evaluated by an experienced pathologist who was blinded to the experiment. All fields in each section were examined for grading of steatosis and necroinflammation according to the Colantoni criteria.^25^ Steatosis was scored as the percentage of parenchymal cells containing fat (microsteatosis or macrosteatosis): 0 = no parenchymal cells containing fat, 1 = 20% of parenchymal cells containing fat, 2 = 20% to 39% of parenchymal cells containing fat, 3 = 40% to 50% of parenchymal cells containing fat, 4 = more than 50% of parenchymal cells containing fat. Inflammation and necrosis were scored based on the number of foci of inflammation and necrosis identified under low‐power field of light microscope: 0 = no inflammation and necrosis, 1 = 1 focus per low‐power field of inflammation and necrosis, 2 = 2 foci per low‐power field of inflammation and necrosis, 3 = 3 or more foci per low‐power field of inflammation and necrosis. 4 = massive inflammation and necrosis. The final score is an average of both steatosis and cell necrosis and inflammation.

### 2.8 TUNEL staining

Hepatic cell death was detected by using the in situ terminal deoxynucleotidyl transferase dUTP nick end labeling (TUNEL) assay (Millipore) according to the manufacturer's instructions. TUNEL‐positive nuclei were visualized by green FITC fluorescence.

### 2.9 Immunohistochemistry

For in vivo fluorescence staining, the liver sample was fixed in 4% paraformaldehyde in 0.1 mol/L PBS solution overnight for 16 hours at 4°C. This was followed by incubation in 30% sucrose solution for 24 hours at 4°C. The liver was then cryosectioned at −20°C into 25‐μm sections and mounted onto slides. Sections were rinsed in 0.1% Triton in PBS (0.1% PBS‐T) and incubated in a blocking solution of 10% normal donkey serum in 0.1% PBS‐T. Sections were washed with PBS and incubated overnight with rabbit caspase‐1 (1:200, ab1872; Abcam, Cambridge, UK), rabbit anti‐activated nuclear factor‐κB (NF‐κB; 1:200; Abcam). mouse anti‐CD68 (1:200; Abcam), mouse anti‐ASC (1:200; Santa Cruz), goat anti‐TLR‐4 (1:200; Santa Cruz), mouse anti‐histone H3 (1:200; Abcam), mouse anti‐NLRP3 (1:200; Abcam), mouse anti‐AIM2 (1:200; Santa Cruz), rabbit anti–HIF‐1α (1:200; Novus),and mouse anti‐VEGF (1:200; Abcam). For in vitro fluorescence staining, cells were fixed in paraformaldehyde in 0.1 mol/L PBS solution. Cells were then incubated in 10% normal donkey serum in 0.1 mol/L PBS Tween‐20 and then incubated overnight with rabbit caspase‐1 (1:200; Abcam), mouse anti‐ASC (1:200; Santa Cruz), followed by secondary antibody for 1 hour. For double‐labeled immunofluorescence, cells and tissue samples were incubated with the first primary antibody overnight, followed by the first secondary antibody, and then the second primary antibody and the second secondary antibody. The slides were counterstained with nuclear dye 4′,6‐diamidino‐2‐phenylindole and mounted with Vectashield mounting medium (Vector Laboratories, Burlingame, CA). Immunofluorescence was quantified by using ImageJ (National Institutes of Health, Bethesda, MA). Ten high‐power fields at ×20 magnification were first photographed by using an AxioCam digital camera (Zeiss, Welwyn Garden City, UK) mounted on an Olympus BX60 microscope (Olympus, Middlesex, UK) with Zeiss KS‐300 software (Zeiss, Welwyn Garden City, UK). The average density per section was calculated (ImageJ). Values were then calculated as percentages of the mean value for naïve controls and expressed as percent fluorescence intensity (FI).

### 2.10 Western blotting

Liver samples were mechanically homogenized in lysis buffer. The lysates were centrifuged, the supernatant was collected, and total protein concentration in the supernatant was quantified according to the Bradford protein assay (Bio‐Rad, Hemel Hempstead, UK). The protein extracts (40 μg/sample) were heated, denatured, and loaded on a NuPAGE 4%‐12% Bis‐Tris gel (Invitrogen) for electrophoresis and then transferred to a polyvinylidene difluoride membrane. The membrane was treated with blocking solution (5% dry milk in TBS with 0.1% Tween‐20) for 2 hours and probed with the following primary antibodies: mouse anti–caspase‐1 p20 (sc‐398715, 1:1000; Santa Cruz), in TBS‐T overnight at 4°C, followed by HRP‐conjugated secondary antibody for 1 hour. The loading control was the constitutively expressed protein glyceraldehyde 3‐phosphate dehydrogenase (1:1000; Abcam). The blots were visualized with use of the enhanced chemiluminescence system (Santa Cruz) and analyzed with the use of GeneSnap (Syngene, Cambridge, UK).

### 2.11 Enzyme‐linked immunosorbent assay

Rat serum and liver tissue IL‐1β, IL‐18 (Rat IL‐1β, IL‐18 ELISA Kit; Invitrogen, Paisley, UK), histone H3 (Histone H3 ELISA kit; LSBio), and VEGF (Rat VEGF Quantikine ELISA Kit; R&D, Abingdon, UK) were measured with use of an ELISA. U937 medium IL‐1β and interferon (IFN)‐γ were assessed by ELISA (Human IL‐1β and IFN‐γ Elisa Kit; Invitrogen).

### 2.12 Kidney and liver function

Blood samples were collected when animals were killed. After centrifugation, asparate aminotransferase (AST) and alanine aminotransferase (ALT) concentrations were measured using AST and ALT Activity Assay Kit (Sigma Aldrich). Serum urea and creatinine concentrations were measured by using an Olympus AU2700 analyzer (Diamond Diagnostics, Watford, UK).

### 2.13 Statistical analysis

All numerical data were expressed as mean ±  SD. Data were analyzed with ANOVA followed by Kruskal–Wallis nonparametric (scoring) or Newman–Keuls (measurement) test for comparisons. A *P* value of <.05 was considered to be of statistical significance.

## 3 RESULTS

### 3.1 IRI in renal graft caused remote liver injury after grafting

**Figure 1:**
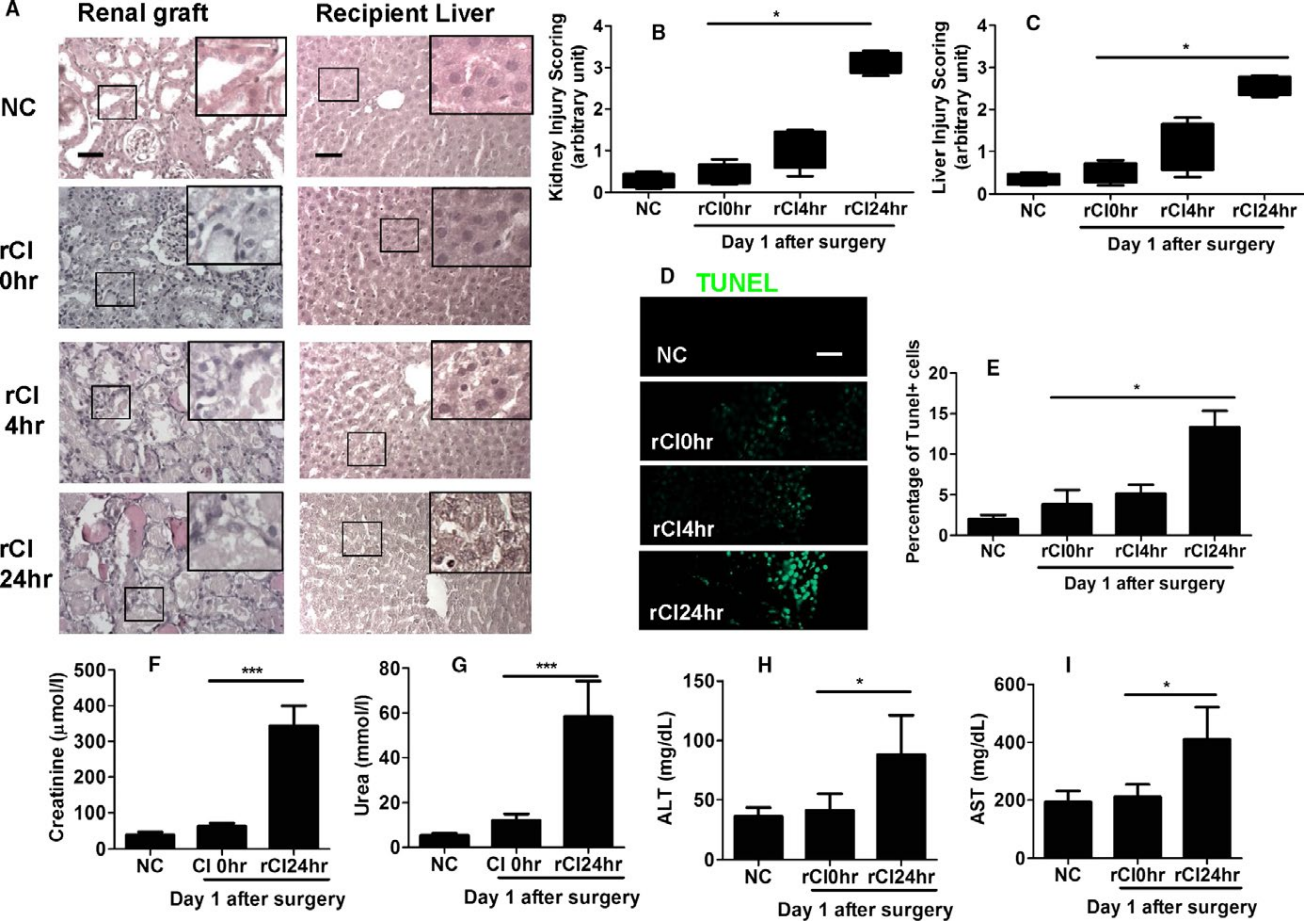
Ischemia–reperfusion injury in the kidney causing liver injury. Renal graft from the Brown–Norway rat donor was stored in 4°C Soltran preservation solution for 0 (live transplantation), 4, or 24 hours (cold ischemia rCI0, rCI4, or rCI24 h), which was subsequently transplanted into Lewis rat recipients. The renal grafts and livers were harvested on day 1 after transplantation, and the contralateral kidney was removed. (A) Histology (hematoxylin–eosin staining) of renal graft and recipient liver. (B) Injury scoring of kidney morphology. (C) Injury scoring of liver morphology. (D) Cell death of liver tissue accessed by TUNEL. (E) Percentage of TUNEL‐positive cells in liver. Serum concentration of (F) creatinine, (G) urea, (H) ALT, and (I) AST in recipient rats. Scale bar: 50 μm. Data expressed as mean ± SD (n = 6) (*P < .05 and ***P < .001). ALT, alanine transaminase; AST, aspartate transaminase; NC, naive control; rCI, renal graft cold ischemia [Color figure can be viewed at http://wileyonlinelibrary.com]

To evaluate the effect of IRI of renal grafts on the liver, renal grafts were stored in cold preservation solution for up to 24 hours and then transplanted into the recipient. There were minimal histologic changes in the liver of recipient with live transplantation. Kidney sections of rats with ischemic injury showed extensive, multifocal, neutrophil‐infiltrated areas of coagulative necrosis scattered throughout the renal parenchyma (Figure 1A,B). Light microscopic evaluation revealed that recipients with cold ischemia for 24 hours had widespread hepatic injury with marked congestion, hepatocyte vacuolization, sinusoidal dilatation, central vein dilatation, Kupffer cell activation, and periportal inflammation (Figure 1A,C). Impaired renal and liver function was also observed in recipients after receiving ischemic renal allografts (Figure 1F‐I), while ischemia–reperfusion in kidney caused significant increase in serum ALT and AST levels compared with sham, indicating a high level of hepatocyte injury. Hepatocyte cell death was also confirmed through TUNEL staining (Figure 1D,E).

### 3.2 Enhanced inflammation was found in remote hepatic injury

**Figure 2:**
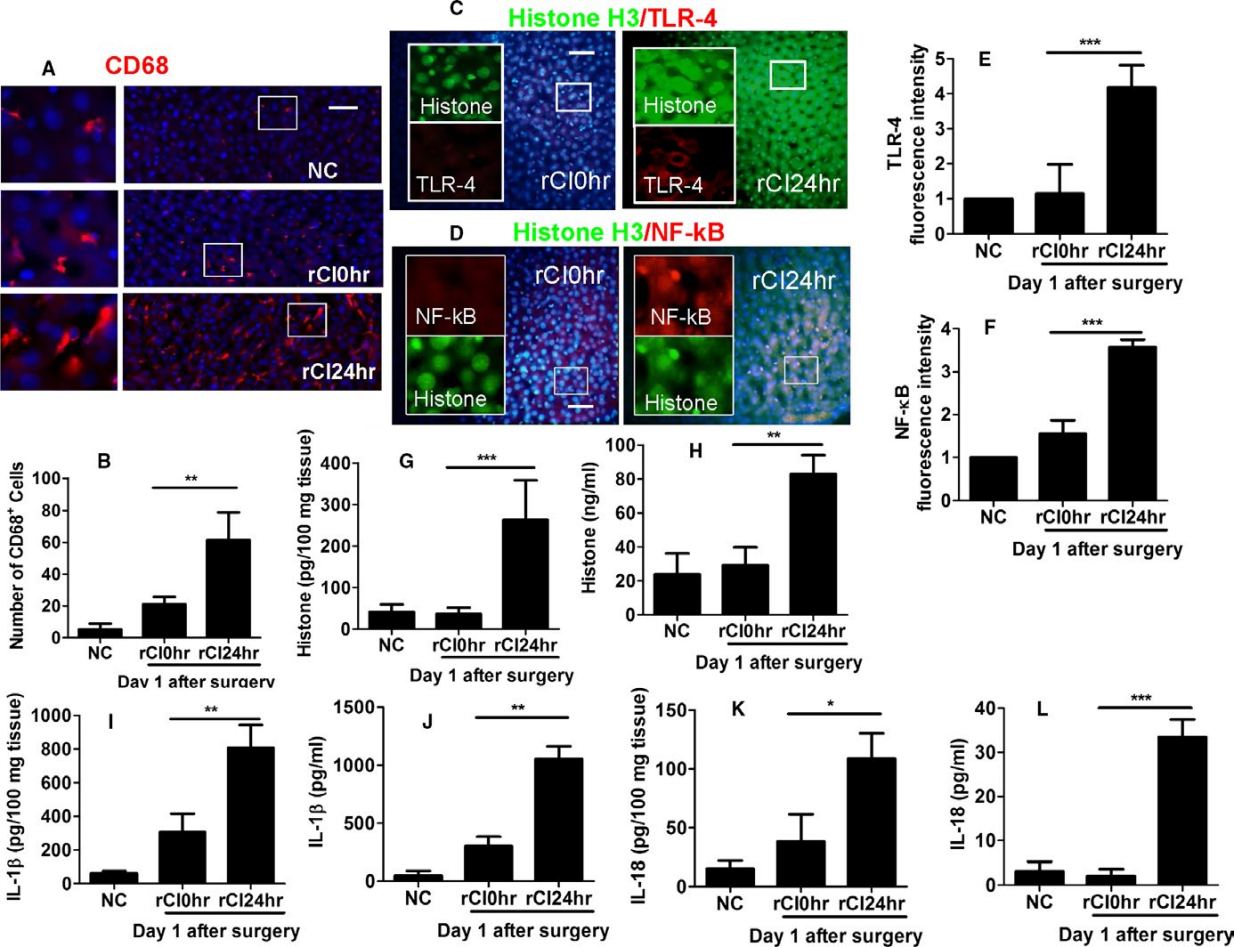
Enhanced inflammation during remote hepatic injury in recipient rats. Renal graft from the Brown–Norway rat donor was stored in 4°C Soltran preservation solution for 24 h (cold ischemia rCI24 h), which was subsequently transplanted into Lewis rat recipients. The livers were harvested on day 1 after transplantation. (A) Immunofluroscence of CD68^+^  (red) macrophages. (B) Number of CD68^+^  macrophages. Dual labeling of (C) histone H3 (green) and TLR‐4 (red) and (D) histone H3 (green) and NF‐κB (red). Fluorescence intensity of (E) TLR‐4 and (F) NF‐κB. Concentration of histone H3 in (G) tissue and (H) serum accessed by ELISA. Concentration of IL‐1β in (I) tissue and (J) serum accessed by ELISA. Concentration of IL‐18 in (K) tissue and (L) serum accessed by ELISA. Scale bar: 50 μm. Data expressed as mean ± SD (n = 6) (*P < .05, **P < .01 and ***P < .001). NC, naive control; rCI, renal graft cold ischemia [Color figure can be viewed at http://wileyonlinelibrary.com]

Renal IRI was associated with a significant increase in CD68^+^ macrophages (Figure 2A,B) by up to 6‐fold compared with naive controls, indicating an increase in infiltrating monocytes or Kupffer cell activation and enhanced inflammation in remote hepatic injury. Furthermore, renal graft IRI was associated with an increase in TLR‐4 (Figure 2C,E) and NF‐κB expression (Figure 2D,F). Through immunoflurocence technique, we observed that histone translocation from the nucleus to cytoplasm occurs in the injury group (Figure 2C,D), indicating release of histone from cells and colocalization with TLR‐4 and NF‐κB. Tissue and serum levels of histone (Figure 2G,H), IL‐1β (Figure 2I,J), and IL‐18 (Figure 2K,L) were evaluated by ELISA, with levels of all 3 markers significantly increased posttransplantation compared with naive controls, indicating increased pyroptosis.

### 3.3 Pyroptosis was induced in remote hepatic injury in recipient with ischemic allografts

**Figure 3:**
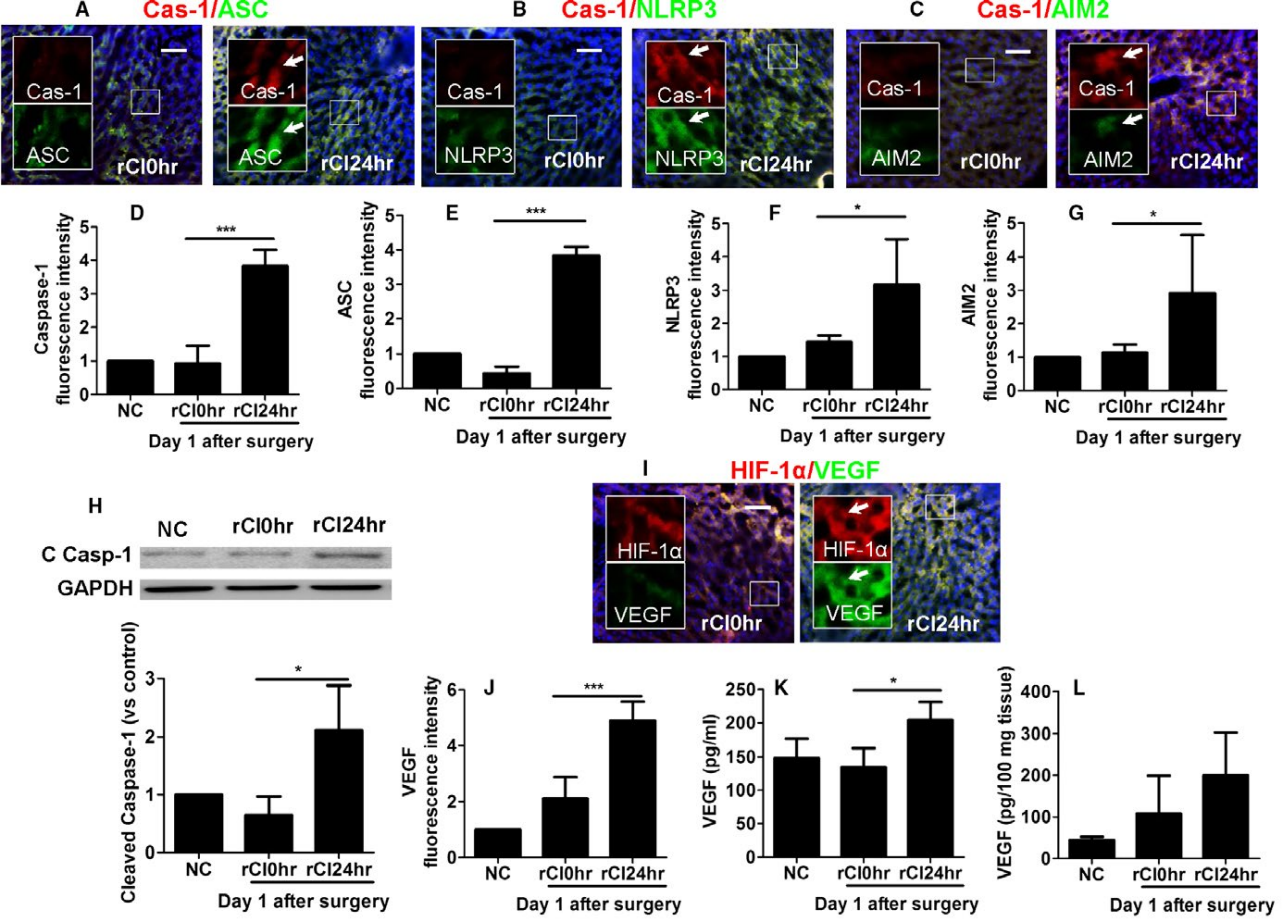
Pyroptosis in remote hepatic injury in recipient with ischemic allografts. Renal graft from the Brown–Norway rat donor was stored in 4°C Soltran preservation solution for 24 h (cold ischemia rCI24 h), which was subsequently transplanted into Lewis rat recipients. Dual labeling of (A) caspase‐1 (red) and ASC (green), (B) caspase‐1 (red) and NLRP3, and (C) caspase‐1 (red) and AIM2 (green). Nuclei were counterstained by DAPI. The fluorescence intensity of (D) caspase‐1, (E) ASC, (F) NLRP3, and (G) AIM2. (H) Expression of cleaved caspase‐1 p20 in liver samples, assessed by Western blot. (I) Dual labeling of HIF‐1α (red) and VEGF (green). (J) Florescence intensity of VEGF. Concentration of VEGF in (K) serum and (L) liver tissue. Scale bar: 50 μm. Data expressed as mean ± SD (n = 6) (*P < .05 and ***P < .001). ASC, caspase recruitment domain; Cas‐1, caspase‐1; C Casp‐1: cleaved caspase‐1; DAPI, 4′,6‐diamidino‐2‐phenylindole; NC, naive control; NLRP3, nucleotide‐binding domain, leucine‐rich repeat containing protein 3; rCI, renal graft cold ischemia. Arrows indicate the colocalization [Color figure can be viewed at http://wileyonlinelibrary.com]

Transplantation of ischemic renal grafts was associated with increased hepatic pyroptosis in remote hepatic injury, indicated by a significant increase in caspase‐1 (Figure 3A,D) and ASC (Figure 3A,E) expression, as well as significant activation of both the NLRP3 (Figure 3B,F) and AIM2 inflammasomes (Figure 3C,G), with colocalization of caspase‐1 with ASC (Figure 3A), NLRP3 (Figure 3B), and AIM2 (Figure 3C). Increased expression of cleaved caspase‐1 was observed (Figure 3H). Furthermore, activation of the HIF‐VEGF system (Figure 3I) was noted, associated with an enhanced expression of VEGF (Figure 3J) by 5‐fold compared with naive controls, with ELISA demonstrating an increased expression of VEGF in both liver tissue and serum (Figure 3K,L). Taken together, these findings indicate the oligomerization of NLRP and AIM2, formation of the NLRP3 and AIM2 inflammasomes, and subsequent recruitment of ASC and pro–caspase‐1, resulting in an increase in hepatic pyroptosis in remote hepatic injury, while activation of the HIF‐VEGF system also occurs after renal graft transplantation.

### 3.4 Histone‐induced pyroptosis in cultured hepatocytes and activation of monocyte

**Figure 4:**
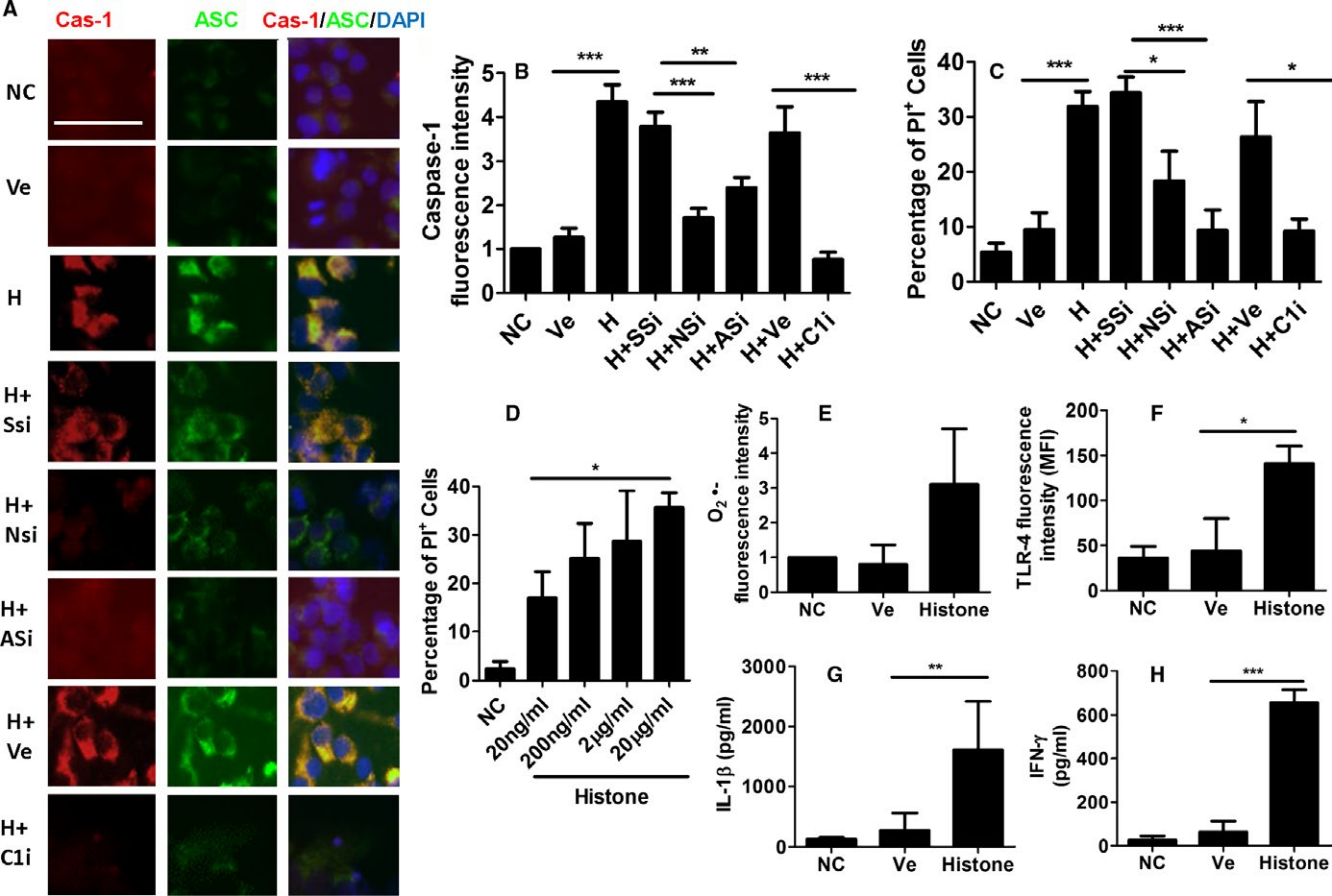
Pyroptosis induced by histone in cultured hepatocytes and activation of monocyte. Some HepG2 cells were treated with histone H3 recombinant protein or PBS vehicle for 24 hours. Some HepG2 cells were treated with human NLRP3 siRNA, AIM2 siRNA, or scrambled siRNA 6 hours before experiments. Some HepG2 cells received caspase‐1 inhibitor Ac‐YVAD‐CHO. Both HepG2 cells and U 937 cells received histone treatment. (A) Dual labeling of caspase‐1 (red) and ASC (green). Nuclei were counterstained with DAPI. Fluorescence intensity of (B) caspase‐1, (C) percentage of HepG2 cell death, assessed by propidium iodide (PI) staining through flow cytometry. HepG2 cells were treated with range of histone H3 recombinant protein (20 ng/mL to 20 μg/mL) for 24 hours. (D) Percentage of HepG2 cell death, assessed by PI staining through flow cytometry. In U937 cells, the fluorescence intensity of (E) superoxide and (F) TLR‐4, medium concentration of (G) IL‐1β and (H) IFN‐γ. Scale bar: 50 μm. Data expressed as mean ± SD (n = 8) (*P < .05, **P < .01 and ***P < .001) [Color figure can be viewed at http://wileyonlinelibrary.com]

Histone‐induced pyroptosis was investigated in cultured HepG2 hepatocytes to determine the effects of extracellular histone in remote hepatic injury, as demonstrated in Figure 4. Our findings indicate that histone H3 treatment is associated with an increase in caspase‐1 (Figure 4A,B), ASC (Figure 4A) expression, and histone dose‐dependent cell death (Figure 4D), suggesting the presence of histone‐induced pyroptosis in hepatocytes. Treatment with *NLRP3* siRNA and *AIM2* siRNA resulted in a significant attenuation of these effects (Figure 4B), indicating a role of both types of inflammasome in the development of histone‐induced pyroptosis. Furthermore, administration of caspase‐1 inhibitor also attenuated these effects (Figure 4B). Cell death was also assessed by PI staining flow cytometry (Figure 4C); treatment with a caspase‐1 inhibitor also reduced the number of PI‐positive cells, indicating that the cell death occurs via pyroptosis. Histone H3–induced activation in cultured U938 monocytes was also assessed, with histone H3 administration being associated with increased reactive oxygen species production (Figure 4E), increased TLR‐4 expression (Figure 4F), and increased IL‐1β (Figure 4G) and IFN‐γ secretion (Figure 4H), indicating monocyte activation and differentiation to macrophages.

### 3.5 Recombinant histone protein treatment exacerbates the remote hepatic injury

**Figure 5:**
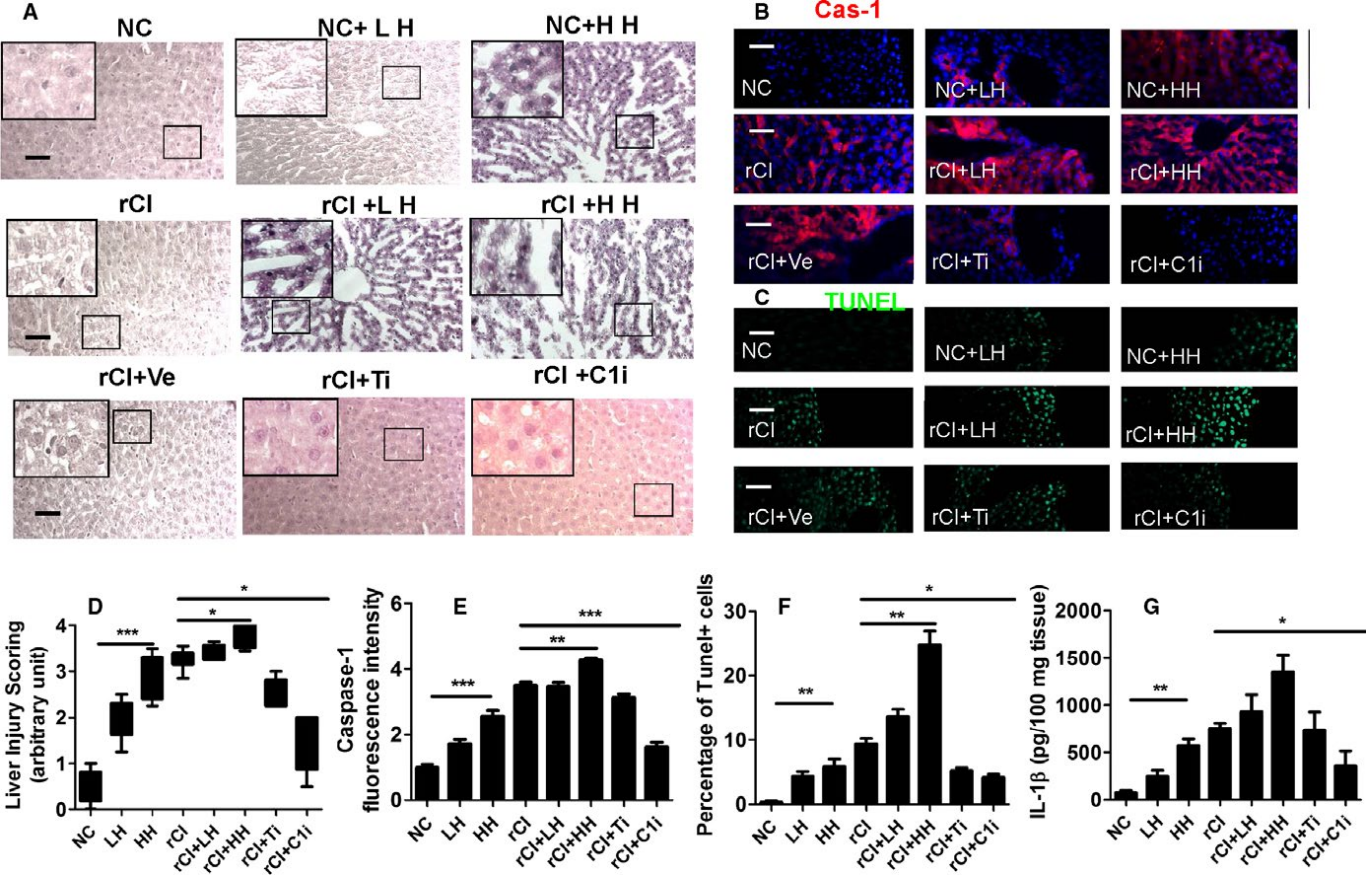
Effects of recombinant histone protein treatment on the remote hepatic injury. Rats with or without transplant surgery were administered with high dose and low dose of histone H3. After transplant surgery, TLR‐4 inhibitor and caspase‐1 inhibitor were administered to the recipient rats. (A) Histology (hematoxylin–eosin staining) of liver tissue. (B) Expression of caspase‐1 accessed by immunohistochemistry. Nuclei were counterstained with DAPI. (C) Cell death accessed by TUNEL (green fluorescence). (D) Injury scoring of liver morphology. (E) Fluorescence intensity of caspase‐1. (F) Percentage of dead hepatocytes. (G) Tissue concentration of IL‐1β. Scale bar: 50 μm. Data expressed as mean ± SD (n = 6) (*P < .05, **P < .01 and ***P < .001). Cas‐1, caspase‐1; DAPI, 4′,6‐diamidino‐2‐phenylindole; HH, high dose of histone H3; LH, low dose of histone H3; NC, naive control; rCI, renal graft cold ischemia; Ti, TLR‐4 inhibitor; C1i, caspase‐1 inhibitor; Ve, vehicle [Color figure can be viewed at http://wileyonlinelibrary.com]

As indicated in Figure 5, recombinant histone H3 protein treatment was found to exacerbate hepatic injury, with both low‐dose and high‐dose histone administration associated with increased hepatic injury (Figure 5A) and increased liver injury score (Figure 5D) by almost 4‐fold. High levels of hepatocyte steatosis and nuclear fragmentation were evident. Caspase‐1 expression (Figure 5B,E), the number of TUNEL‐positive cells (Figure 5C,F), and tissue IL‐1β concentration (Figure 5G) are consistent with histologic findings, with TLR‐4 inhibitor or caspase‐1 inhibitor administration associated with a reduction of these effects. Liver injury score (Figure 5D) was attenuated with the administration of TLR‐4 inhibitor or caspase‐1 inhibitor. These findings indicate that TLR‐4 blocking or capase‐1 inhibition may be a potential therapeutic option to reduce the effects of histone‐induced pyroptosis in remote hepatic injury associated with IRI in rat renal allografts.

### 3.6 VEGF‐mediated cytoprotection in the remote liver injury

**Figure 6:**
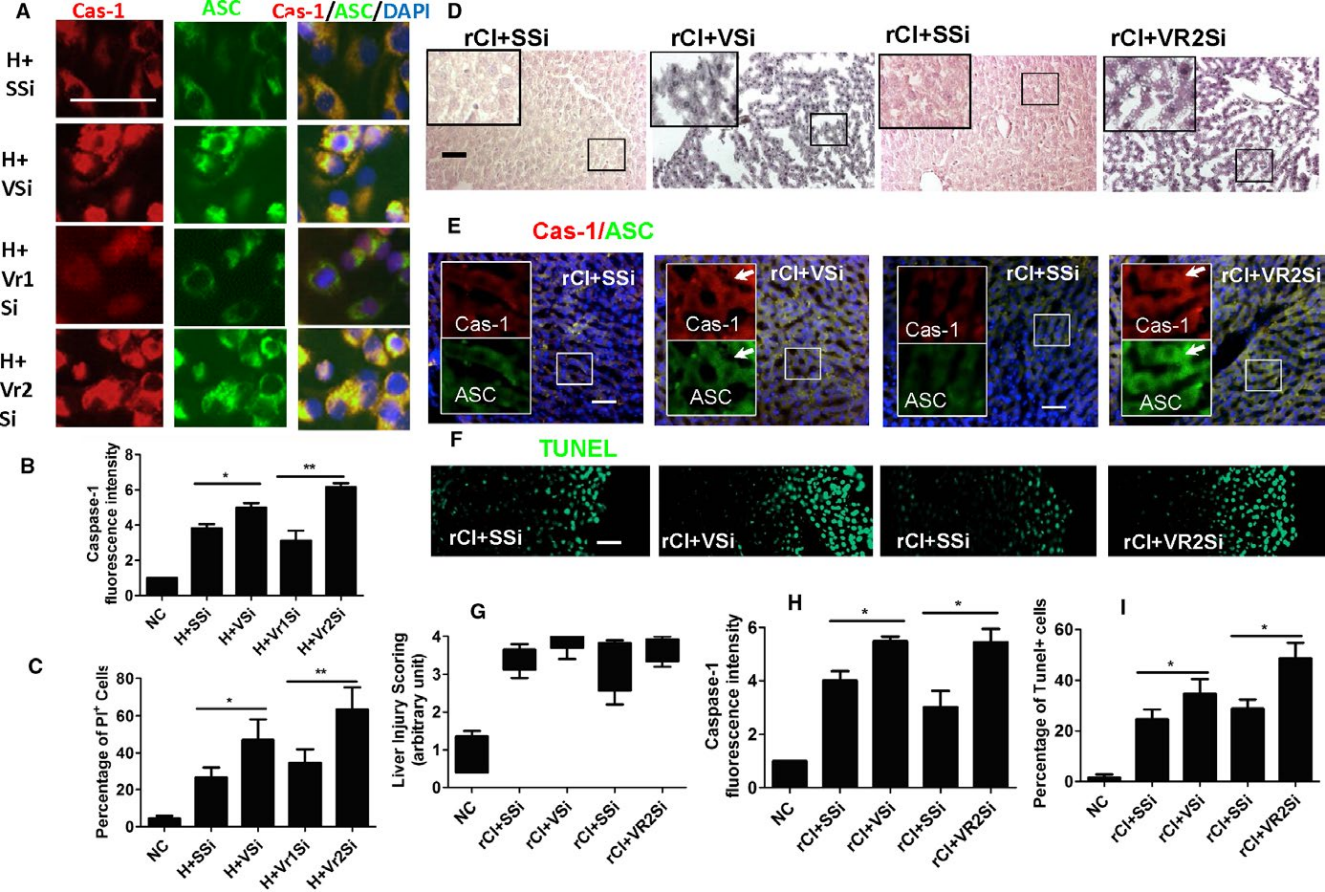
Cytoprotection mediated by vascular endothelial growth factor in the remote liver injury. Cultures of HepG2 cells were treated with VEGF siRNA, VEGFR1 siRNA, VEGFR2 siRNA, or scrambled siRNA 6 hours before histone H3 treatment. In rat transplant recipient, VEGF siRNA or VEGF R2 siRNA or scrambled SiRNA was also administered to recipient rats. (A) Dual labeling of caspase‐1 (red) and ASC (green). All nuclei were counterstained with DAPI. (B) Florescence intensity of caspase‐1. (C) Percentage of dead cells. (D) Histology (hematoxylin–eosin staining) of liver tissue. (E) Dual labeling of caspase‐1 (red) and ASC (green). Nuclei were counterstained with DAPI. Arrows indicate the colocalization. (F) Cell death accessed by TUNEL. (G) Scoring of liver injury. (H) Fluorescence intensity of caspase‐1. (I) Percentage of cell death. Scale bar: 50 μm. Data is expressed as mean ± SD (n = 6) (*P < .05 and **P < .01). ASC, caspase recruitment domain; Cas‐1, caspase‐1; DAPI, 4′,6‐diamidino‐2‐phenylindole; H, Histone H3; rCI, renal graft cold ischemia; SSi, scrambled siRNA; VSi, VEGF siRNA; Vr1Si, VEGFR1 siRNA; Vr2Si, VEGFR2 siRNA; Ve, vehicle [Color figure can be viewed at http://wileyonlinelibrary.com]

The capability for VEGF to attenuate pyroptosis in remote liver injury was assessed. In cultured hepatocytes treated with histone and in liver tissue with remote injury, expression of caspase‐1 (Figure 6A,B,E) and ASC (Figure 6A,E) was increased, while treatment with *VEGF* siRNA enhanced the expression of these markers, thus indicating hepatic protection. VEGF receptors 1 and 2 siRNA, blocking the expression of receptors 1 and 2, respectively, were administered. Unlike receptor 1, blocking receptor 2 significantly enhanced the expression of caspase‐1 (Figure 6A,B) and ASC (Figure 6A) and increased the percentage of PI‐positive cells (Figure 6C), suggesting that VEGF acts via receptor 2. After transplantation, treatment with VEGF siRNA or VEGF receptor 2 siRNA exacerbated hepatic injury (Figure 6G), enhanced caspase‐1 (Figure 6B,H) and ASC expression (Figure 6E) and increased the number of TUNEL‐positive cells (Figure 6I). This indicates that blocking the VEGF signaling pathway would suppress the VEGF protective effects.

### 3.7 Recombinant VEGF protein confers cytoprotection against histone‐induced pyroptosis

**Figure 7:**
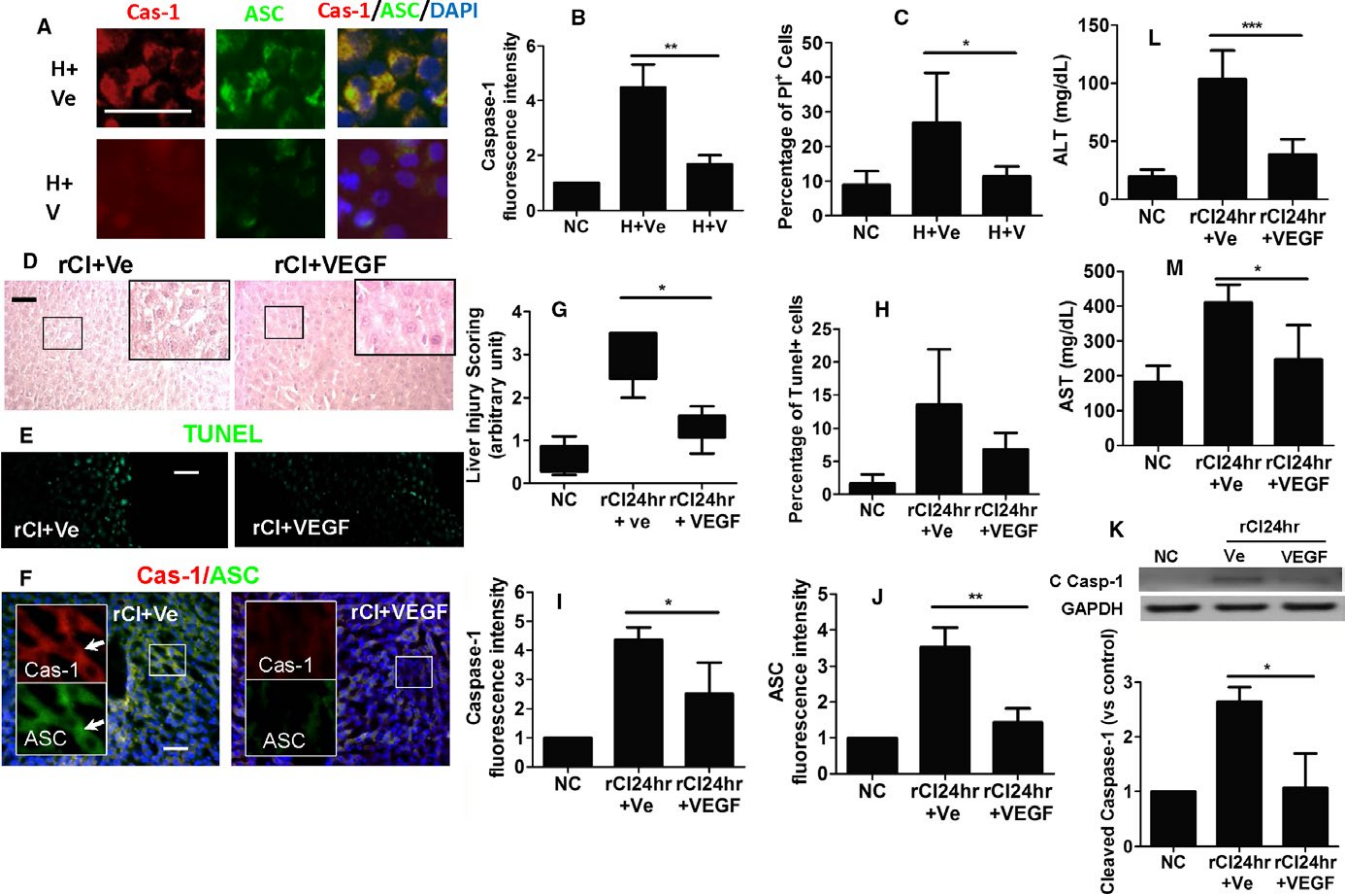
Cytoprotection mediated by recombinant VEGF protein against histone‐induced pyroptosis. In vitro, cultures of HepG2 cells were treated with histone H3 recombinant protein, VEGF recombinant protein, or PBS for 24 hours. (A) Dual labeling of caspase‐1 (red) and ASC (green) in HepG2 cells. Nuclei were counterstained with DAPI. (B) Fluorescence intensity of caspase‐1 in HepG2 cells. (C) Percentage of dead cells. Renal graft from the Brown–Norway rat donor was stored in 4°C Soltran preservation solution for 24 hours (cold ischemia rCI24 h), which was subsequently transplanted into Lewis rat recipients. (D) Histology (hematoxylin–eosin staining) of liver tissue. (E) Liver hepatocyte cell death accessed by TUNEL. (F) Dual lablling of caspase‐1 (red) and ASC (green) in liver tissue. Nuclei were counterstained with DAPI. Arrows indicate the colocalization. (G) Scoring of liver injury. (H) Percentage of necrotic cells. Fluorescence intensity of (I) caspase‐1 and (J) ASC in liver tissue. (K) Expression of cleaved caspase‐1 p20 in liver samples, assessed by Western blot. Concentration of liver enzymes (L) ALT and (M) AST. Scale bar: 50 μm Data expressed as mean ± SD (n = 6) (*P < .05, **P < .01, and ***P < .001). ASC, caspase recruitment domain; Cas‐1, caspase‐1; C Casp‐1, cleaved caspase‐1; DAPI, 4′,6‐diamidino‐2‐phenylindole; H, Histone H3; rCI, renal graft cold ischemia; Ve, vehicle; V, VEGF recombinant protein [Color figure can be viewed at http://wileyonlinelibrary.com]

Recombinant VEGF protein was found to confer cytoprotection against pyroptosis, as demonstrated in Figure 7. After being challenged with histone, HepG2 cells, when treated with VEGF, exhibited reduced caspase‐1 (Figure 7A,B) and ASC expression (Figure 7A), as well as a reduced number of PI‐positive cells (Figure 7C), indicating VEGF‐mediated cytoprotection. After transplantation, animals receiving recombinant VEGF demonstrated improved histologic morphology (Figure 7D), as well as a reduced number of TUNEL‐positive cells (Figure 7E,H). Furthermore, administration of recombinant VEGF was also associated with significantly reduced expression of caspase‐1 (Figure 7F,I) and ASC (Figure 7F,J) by one‐half and two‐thirds, respectively, as well as an improvement in liver injury score (Figure 7G). The expression of cleaved caspase‐1 was also reduced by this treatment (Figure 7K). Recombinant VEGF also caused a significant improvement in hepatic function, as evidenced by a reduction in both ALT and AST levels in Figure 7L and M, respectively. These findings indicate that VEGF may be a potential therapeutic agent to attenuate pyroptosis in hepatic injury and reduce the deleterious effects of remote liver injury associated with renal graft IRI.

### 3.8 Recombinant VEGF protein attenuates inflammation in remote hepatic injury

**Figure 8:**
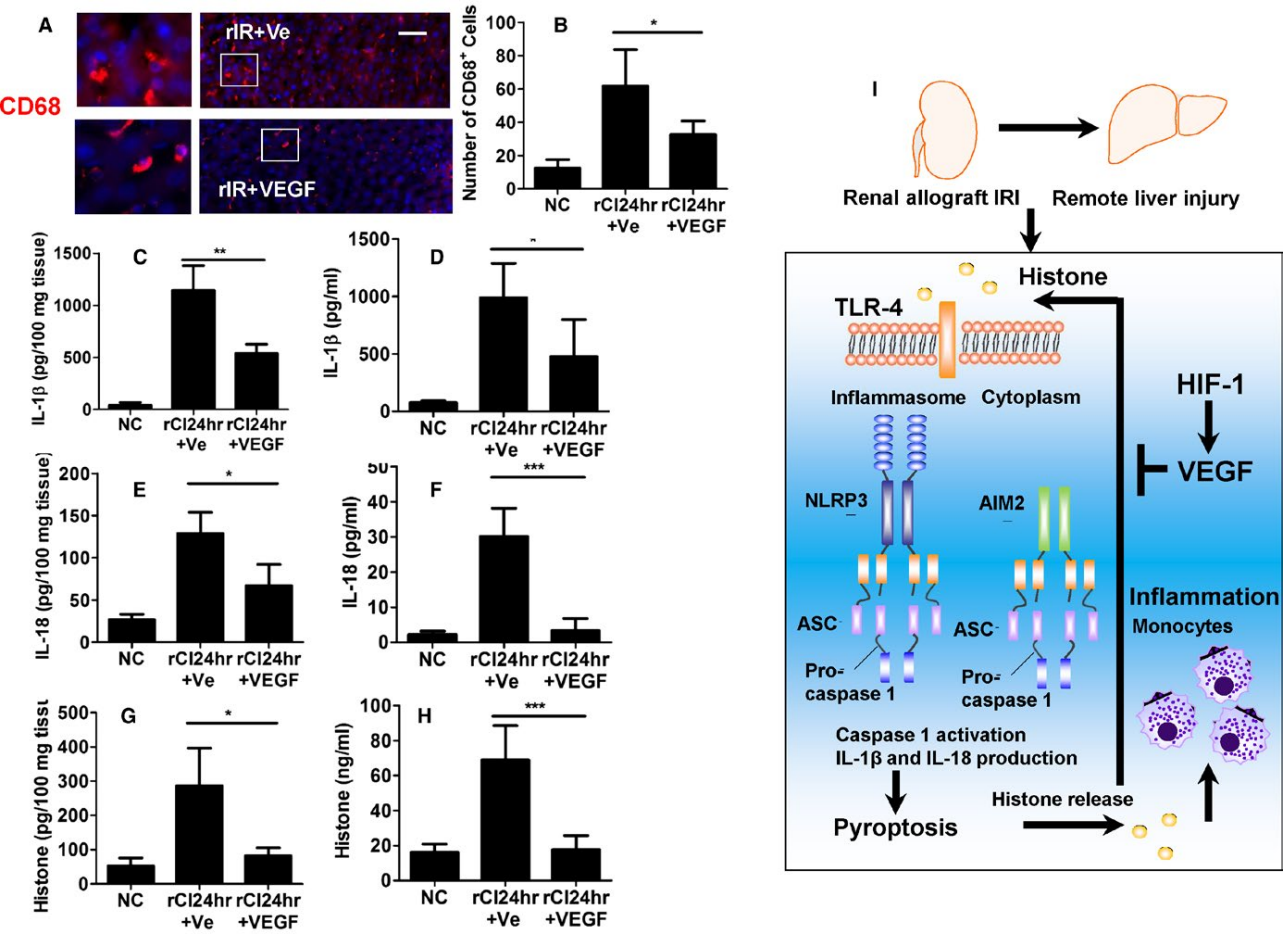
Reduced inflammation by recombinant VEGF protein in remote hepatic injury. Renal graft from the Brown–Norway rat donor was stored in 4°C Soltran preservation solution for 24 hours (cold ischemia rCI24 h), which was subsequently transplanted into Lewis rat recipients. The recipient rats were administered with VEGF recombinant protein intravenously. (A) Immunofluroscence labeling of CD68^+^  macrophages. (B) Number of CD68^+^  cells. Concentration of IL‐1β in (C) tissue and (D) serum, accessed by ELISA. Concentration of IL‐18 in (E) tissue and (F) serum, accessed by ELISA. Concentration of histone H3 in (G) tissue and (H) serum, accessed by ELISA. (I) Proposed mechanisms of pyroptosis induced by histone in remote liver injury after transplantation; extracellular histone binds to TLR‐4 receptor, activating both NLRP3 and AIM 2 inflammasome. The production of IL‐1β and IL‐18 is increased and cells die by pyroptosis. In addition, pyroptosis enhanced activation of monocytes through histone release. VEGF protects against remote liver injury through attenuating the pyroptosis. Scale bar: 50 μm. Data expressed as mean ± SD (n = 6) (*P < .05, **P < .01 and ***P < .001). Ve, vehicle [Color figure can be viewed at http://wileyonlinelibrary.com]

The administration of VEGF protein is found to be associated with a reduction in inflammation‐induced hepatic injury. Administration of VEGF causes significant reduction in the number of CD68^+^  cells (Figure 8A,B), as well as reduced production of pyroptosis‐related cytokines, IL‐1β (Figure 8C,D), and IL‐18 (Figure 8E,F), in both liver tissue and serum. ELISA indicates reduced tissue and serum concentration of histone (Figure 8G,H), suggesting an attenuation of histone release after VEGF protein administration. Overall, these findings indicate that treatment with VEGF protein results in a reduction in hepatic inflammation, macrophage infiltration, pyroptosis‐mediated hepatocyte injury, and histone release (Figure 8I).

## 4 DISCUSSION

In the present study, we demonstrated for the first time the progressive increase in histone release and induced pyroptosis during remote hepatic injury in a rat model of renal allograft transplantation. Blocking the histone–TLR‐4 pathway prevented the hepatic pyroptosis caused by renal allograft IRI. In addition, VEGF was found to mitigate the histone‐induced development of pyroptosis and subsequent hepatic inflammation and could serve as a therapeutic target within these pathophysiologic processes.

It is interesting to note that Nakazawa et al. recently investigated the role of circulating neutrophil extracellular traps and histones in remote organ injury after warm renal IRI.^26^ The authors investigated the systemic effects of AKI on remote organs and found that tubular necrosis and neutrophil extracellular trap formation promote kidney injury and remote organ damage due to the systemic release of proinflammatory cytokines and histones. In addition, AKI results in increased levels of circulating histones, with concurrent neutrophil infiltration in the liver, lungs, brain, and heart. Correspondingly, pretreatment with antihistone IgG results in the suppression of neutrophil extracellular trap formation and an attenuation in subsequent renal injury. When taking our novel findings in the context of Nakazawa et al.'s results, the combined blocking of cytokine and histone release, in combination with VEGF administration, may possess additive protective effects and be potential future therapeutic avenues to limit remote liver injury associated with renal allograft IRI.

The onset of liver injury after IRI in renal allografts appeared in early stages of kidney injury and was associated with increased levels of proinflammatory cytokine and activated oxidative stress. Histones are the proteins in chromatin that play an important role in controlling gene expression.^27^ There are 2 kinds of histones: core histones (including H2A, H2B, H3, and H4) and linker histones (namely, H1 and H5).^27^ Despite their vital role in gene regulation, histones in the extracellular space can cause inflammation and organ damage as reported in liver, lung, and kidney injury.^10^, ^12^, ^28^ When necrosis takes place, the cell membrane ruptures and the cellular content, also known as damage‐associated molecular patterns (DAMPs) including histones, would be released, causing inflammatory response.^29^, ^30^ The release of histones may also be associated with the formation of neutrophil extracelluar traps, which has shown to cause cell death in the lungs and endothelium.^31^ Allam et al. showed that histone is released and causes inflammatory response in renal tubular epithelial injury, by binding with the TLR‐4 and TLR‐2.^10^ Histone has been shown to be closely associated with the NLRP3 inflammasome. The activation of NLRP3 leads to the assembly of the NLRP3 inflammasome, which includes pro–caspase‐1, resulting in the production of proinflammatory cytokines IL‐1β. NLRP3 inflammasome is formed after the oligomerization of NLRP3 and subsequent recruitment of ASC and pro–caspase‐1.^32^ On activation of NLRP3, ASC proteins assemble into fiberlike structures; this culminates in the production of a large protein aggregate that amplifies the activation of caspase‐1.^33^ The NLRP3 inflammasome is activated in response to a variety of infectious stimuli or to cellular stress caused by various sterile danger signals, including histone.^32^ The inflammasome induces the release of IL‐1β, IL‐18, which exacerbate the inflammation.^33^, ^34^ Pyroptosis is a caspase‐1–dependent programmed cell death, which features cell swelling, rapid plasma membrane rupture, and release of proinflammatory intracellular contents.^35^ Given this growing body of evidence indicating a diverse role for caspase‐1 in inflammation, we hypothesized that it would play a significant role in the setting of remote hepatic injury. Increased proinflammatory cytokine profile and increased hepatocellular death were found in rats with remote hepatic injury. These findings shed new light on the role of caspase‐1 in the setting of hepatic injury and may lead to new therapeutic approaches for patients with remote liver injury.

It is important to note that while histones have been shown to stimulate pyroptosis in hepatocytes, histones also activate other cell types, such as the NLRP3 inflammasome in Kupffer cells, during liver IRI.^15^ There is accumulating evidence from murine studies that indicates the critical role of endogenous ligands, such as histones and high mobility group box 1, in mediated hepatic IRI via TLR‐9/MyD88 signaling pathways.^12^, ^36^ During liver IRI, endogenous extracellular histones activate the NLRP3 inflammasome via TLR‐9 activation, while resident liver Kupffer cells possess an integral role in histone‐mediated activation of the NLRP3 inflammasome. Kupffer cells promote an innate inflammatory response after IRI, which results in the recruitment and infiltration of a multitude of proinflammatory cells, including inflammatory monocytes and neutrophils.^15^ Extracellular histones have previously been shown to function as DAMPs, thus facilitating hepatic IRI by stimulating the TLR‐9 signaling pathway.^12^ In the previous studies, an increased level of histone was toxic to liver and cause liver injury.^14^ We have also demonstrated that naive animal and recipient animal, when receiving both low and high doses of histone, had significant liver damage, and blocking the its receptor TLR‐4 through inhibitor reduced the injury; all these data indicated that the remote injury was effectively caused by extracellular histones. In addition, it is difficult to distinguish histone release from either kidney or liver, or both. Therefore, it remains an open question whether histones released from kidney are a primary trigger for liver injury or other renal failure–related factors (eg, toxins trigger histone release from liver cells and in turn further exacerbate the injury). This warrants further investigation in future studies.

While it was initially believed that VEGF receptors were solely confined to the vascular endothelium, hence the name of the protein family, the presence of VEGF receptors has been acknowledged in a variety of cell types, including epithelial cells.^37^ As a result, VEGF is capable of eliciting its effects on both epithelial and endothelial surfaces. VEGF‐A binds with a high affinity to both VEGF‐R1 and VEGF‐R2, with VEGF‐R2 undergoing more potent tyrosine phosphorylation after ligand activation, while VEGF‐B solely interacts with VEGF‐R1.^38^ Although VEGF‐R1 has shown a higher affinity to VEGF, approximately 10‐fold higher than VEGF‐R2, VEGF‐R2 is considered a significant positive mitogenic signal transducer due to its strong kinase activity in comparison to VEGF‐R1.^39^ Various studies have demonstrated that VEGF‐R2 upregulation promotes organoprotection. Activation of the VEGF‐R2 pathway has been shown to mediate lung protection against oxidant‐induced acute lung injury.^40^ Furthermore, the lungs of patients with sepsis, which is considered an important trigger of acute lung injury, have been shown to possess a significantly lower level of VEGF‐R2,^41^ while observational studies of lung injury in humans demonstrate a significant reduction in intrapulmonary VEGF during the early stages of acute respiratory distress syndrome.^42^ Our findings demonstrate that VEGF has the potential to induce organoprotective effects, possibly by activating various signaling cascades. Inhibition of VEGF during reperfusion of the highly ischemic allografts, through either *VEGF* siRNA or *VEGF‐R2* siRNA, exacerbates the hepatic injury observed. These processes may, at least in part, explain the mechanism by which VEGF attenuates extracellular histone‐induced pyroptosis in remote hepatic injury.

There are limitations on our studies. First, the exact resource of extracellular histones after renal cold ischemia–reperfusion is unclear; they could be released from kidney graft or liver, or both. Second, our study demonstrated that VEGF treatment attenuates liver damage by reducing histone release from pyroptotic hepatocytes. The expression level of VEGF and the potential effects of VEGF treatment, including siRNA treatment, on kidney graft remain unknown. This certainly requires further investigation. Third, recombinant histone has been shown to exacerbate hepatic injury,^12^, ^13^ while histone neutralization has been shown to be a potential therapeutic avenue to attenuate its hepatotoxic effects. Recent studies have demonstrated that nuclear histone proteins are closely associated with the upregulation of DAMPs, including DNA and HMGB‐1, which are responsible for contributing to hepatic IRI,^12^ as well as promoting cytotoxicity via TLR‐9 and MyD88 pathways.^12^, ^15^ The administration of neutralizing antibodies to extracellular histones (anti‐H3 and anti‐H4 antibodies) confers significant protection after hepatic IRI.^12^ This is thought to occur due to the attenuation of tissue tumor necrosis factor‐α and IL‐6. Further investigation into the hepatoprotective effects of histone neutralization after hepatic IRI is warranted. Finally, human hepatocellular carcinoma cell line was used in in vitro study. It is very different in cell phenotype compared with primary hepatocytes, which are considered to be used for future study.

Patients with AKI often have liver dysfunction and may be associated with higher mortality.^4^, ^6^ Although the effect of renohepatic crosstalk in renal graft recipients remains fully elucidated, our study indicates the role of circulating histones and their inflammatory effect on the liver. Extracellular histones, therefore, may be the target for therapy or prophylaxis against liver damage in kidney transplant patients.

In conclusion, our data suggest that a substantial release of histone in recipient after receiving ischemic renal allografts leads to remote hepatic injury during early the postoperative period and the protective effects of VEGF through blocking histone‐induced pyroptosis in the hepatic remote injury.

## DISCLOSURE

The authors of this manuscript have no conflicts of interest to disclose as described by the *American Journal of Transplantation*.
